# Posttransplant cyclophosphamide versus antithymocyte globulin in patients with cardiovascular comorbidity undergoing allogeneic hematopoietic cell transplantation for acute myeloid leukaemia in first complete remission from unrelated donors: a retrospective matched-pair analysis from the ALWP of the EBMT

**DOI:** 10.1038/s41409-025-02766-0

**Published:** 2025-12-03

**Authors:** Jan Vydra, Allain-Thibeault Ferhat, Nicolaus Kröger, Tobias Gedde-Dahl, Matthias Eder, Thomas Schroeder, Urpu Salmenniemi, Régis Peffault de Latour, Jakob Passweg, Ibrahim Yakoub-Agha, Alessandro Rambaldi, Robert Zeiser, Matthias Stelljes, Kristina Carlson, Cristina Castilla-Llorente, Alexandros Spyridonidis, Bipin Savani, Fabio Ciceri, Mohamad Mohty

**Affiliations:** 1 Institute of Hematology and Blood Transfusion, Prague, Czech Republic; 2 EBMT Paris study office, Department of Haematology, Saint Antoine Hospital; Sorbonne University, Paris, France; 3 University Medical Center Hamburg, Hamburg, Germany; 4 Oslo University Hospital, Rikshospitalet Oslo, Oslo, Norway; 5 Hannover Medical School, Hannover, Germany; 6 University Hospital Essen, Essen, Germany; 7 HUS Comprehensive Cancer Center, Helsinki, Finland; 8 Saint-Louis Hospital, BMT Unit Paris, Paris, France; 9 University Hospital Basel, Basel, Switzerland; 10 CHU de Lille, Univ Lille, INSERM U1286, Infinite, Lille, France; 11 Department of Oncology and Hematology, University of Milan, Milan, Italy; 12 University of Freiburg, Freiburg, Germany; 13 University of Muenster, Muenster, Germany; 14 University Hospital Uppsala, Uppsala, Sweden; 15 Gustave Roussy Cancer Campus, Villejuif, France; 16 Hematology, BMT and Institute of Cellular Therapy, University of Patras, Patras, Greece; 17 Vanderbilt University Medical Center, Nashville, TN USA; 18 Ospedale San Raffaele, Haematology and BMT, Milano, Italy; 19 Sorbonne University, Department of Haematology, Saint Antoine Hospital, Patras, France

**Keywords:** Stem-cell research, Health services

## Abstract

We retrospectively analyzed data from the EBMT registry on patients with pretransplant comorbidities associated with cardiovascular risk. Patients who underwent first allogeneic hematopoietic cell transplantation for acute myeloid leukemia in first complete remission between 2010 and 2022 from unrelated donors using post-transplant cyclophosphamide (ptCy) or anti-thymocyte globulin (ATG)-based graft-versus-host disease prophylaxis with a history of cardiac disease, arrhythmia, diabetes, obesity or cerebrovascular disease according to the HCT-specific comorbidity index were included. We performed a matched-pair analysis using a propensity score. After matching, 432 patients were included: 313 in the ATG group and 119 in the ptCy group. At 2 years, overall survival was 67.5% (95% CI 61–73.2) and 68.6% (95% CI 56.7–77.8); leukemia-free survival was 60.4% (95% CI 53.8–66.4) and 62.6% (95% CI 50.4–72.6); relapse incidence was 22.1% (95% CI 17–27.7) and 23.2% (95% CI 14.3–33.4); non-relapse mortality was 17.5% (95% CI 13.1–22.4) and 14.1% (95% CI 7.5–22.8), respectively. In conclusion, our study suggests that the use of ptCY for GVHD prophylaxis in patients with preexisting comorbidities associated with cardiovascular risk yields long-term outcomes comparable to those observed with ATG-based approaches.

## Introduction

High-dose cyclophosphamide is associated with a risk of acute cardiac toxicity, which is dose-dependent. While mild cases generally resolve with supportive care, severe toxicity can be fatal. Post-transplant cyclophosphamide (ptCy) has emerged as the *de facto* standard for graft-versus-host disease (GVHD) prophylaxis following T cell–replete mismatched donor transplantation and is increasingly employed in human leukocyte antigen (HLA)-matched donor settings, where it has demonstrated outcomes comparable to those of anti-thymocyte globulin (ATG)–based prophylaxis [1–3].

Patients with a history of cardiovascular disease may be at increased risk for adverse outcomes when exposed to cardiotoxic therapies. Therefore, we conducted a retrospective analysis of transplant outcomes in patients with preexisting comorbidities associated with cardiovascular risk, who underwent allogeneic hematopoietic cell transplantation (allo-HCT) from unrelated donors while in first complete remission for acute myeloid leukemia (AML), receiving either ATG- or ptCy-based GVHD prophylaxis. Our aim was to evaluate whether an ATG-based strategy is safer in this group of patients compared with post-transplant cyclophosphamide.

## Subjects and methods

Patients who underwent a first allo-HCT for AML in first complete remission between 2010 and 2022 in one of the European Society for Blood and Marrow Transplantation (EBMT) centers, from HLA 10/10 matched unrelated donors or HLA 9/10 mismatched unrelated donors were included in the study. Patients had to have one or more pretransplant comorbidities associated with cardiovascular risk identified by HCT-specific comorbidity index (HCT-CI) score (cardiac comorbidity, arrhythmia, cerebrovascular disease, obesity, or diabetes) and have received ATG- or ptCY-based GVHD prophylaxis and a conditioning regimen of known intensity that did not include cyclophosphamide or total body irradiation.

The EBMT database contains prospectively collected data on all consecutive transplants performed in member centers. These centers obtain informed consent according to the local regulations applicable at the time of transplantation in order to report pseudonymized data to the EBMT. The study was conducted in accordance with the Declaration of Helsinki guidelines.

A propensity score-based matched-pair analysis was performed to minimize bias arising from baseline differences between patients receiving ATG or ptCY, as well as to address the imbalance in group sizes. Matching was conducted based on the following covariates: stem cell source, patient age at HCT, year of transplantation, donor type, Karnofsky performance score, transplant conditioning intensity score, female donor to male recipient combination, and AML cytogenetic classification. Propensity score matching was conducted at a 3:1 ratio with a caliper width of 0.2. A standardized mean difference less than 0.1 after matching was required to confirm adequate balance. All patients who received ptCY were retained in the analysis due to the smaller size of this cohort.

The primary endpoint was overall survival (OS), and secondary endpoints were non-relapse mortality (NRM), leukemia-free survival (LFS) and relapse incidence (RI). NRM was defined as death without evidence of relapse, OS was defined as time to death from any cause, and LFS as time being alive without evidence of relapse. Outcomes were analyzed using the Kaplan-Meier method, cumulative incidence estimators and univariate Cox models to estimate hazard ratios and statistical significance. NRM and RI were competing risks. Statistical analyses were performed with SPSS 27.0 (SPSS Inc., Chicago, IL, USA) and R version 4.2.1 (R Development Core Team, Vienna, Austria, URL: https://www.R-project.org/).

## Results

1256 patients were identified: 1127 who received ATG and 129 who received ptCy-based GvHD prophylaxis. After propensity score pair-matching, 432 patients were analyzed, 313 in the ATG group and 119 in the ptCy group. Standardized mean difference after pair-matching was <0.10 for all parameters. Patient characteristics of the final dataset are summarized in Table 1.

**Table 1:** Patient characteristics of the final dataset.

	ATG^a^	ptCy^a^	*P* value^b^
	*n* = 313	*n* = 119	
Year of HCT, median (IQR)	2020 (2010, 2022)	2021 (2015, 2022)	0.31
Age, years, median (IQR)	59.9 (51.8, 66.0)	60.9 (52.2, 65.8)	0.70
Sex of patient			0.97
Female	119 (38%)	45 (38%)	
Male	194 (62%)	74 (62%)	
Karnofsky performance score			0.5
≥ 90	235 (75%)	93 (78%)	
<90	78 (25%)	26 (22%)	
Cytogenetic AML classification			0.14
Favorable	14 (4.8%)	1 (1.0%)	
Intermediate	205 (70%)	72 (69%)	
Adverse	74 (25%)	32 (30%)	
Missing	20	14	
Secondary AML	47 (15%)	25 (21%)	0.14
Transplant conditioning intensity score			0.76
1–2	92 (29%)	35 (29%)	
2.5–3.5	172 (55%)	62 (52%)	
4–6	49 (16%)	22 (18%)	
Conditioning regimen			0.22
TBF-based	37 (12%)	24 (20%)	
TreoFlu-based	81 (26%)	27 (23%)	
BuFlu-based	137 (44%)	51 (43%)	
FluMel-based	51 (16%)	14 (12%)	
Other	7 (2.2%)	3 (2.5%)	
Myeloablative regimen	180 (58%)	67 (57%)	0.84
Graft source			0.91
Bone marrow	15 (4.8%)	6 (5.0%)	
Peripheral blood	298 (95%)	113 (95%)	
Type of donor			0.21
MUD (10/10)	86 (27%)	40 (34%)	
MMUD (9/10)	227 (73%)	79 (66%)	
Donor age at HCT, median (IQR)	27.6, (23.3, 35.1)	28.8, (23.8, 35.1)	0.57
Female donor to male patient	46 (15%)	18 (15%)	0.91
HCT-CI			0.057
1–2	149 (53%)	48 (42%)	
≥ 3	134 (47%)	66 (58%)	
Missing	30	5	
HCT-CI comorbidities			
Diabetes	80 (26%)	32 (27%)	0.81
Cardiac	122 (39%)	43 (36%)	0.54
Arrhythmia	52 (17%)	21 (18%)	0.82
Cerebrovascular	29 (10%)	12 (10%)	0.83
Heart valve disease	40 (13%)	14 (12%)	0.77
Obesity	62 (20%)	35 (29%)	0.034

*P* values are reported to illustrate differences in unmatched variables and to confirm that propensity score matching yielded comparable cohorts for matched variables.

*TBF* thiotepa, busulfan, fludarabine, *TreoFlu* treosulfan, fludarabine, *BuFlu* busulfan, fludarabine, *FluMel* fludarabine, melphalan, *MUD* matched unrelated donor (10/10 HLA match), *MMUD* mismatched unrelated donor (9/10 HLA match), *HCT* hematopoietic cell transplantation, *HCT-CI* Hematopoietic Cell Transplantation-Comorbidity Index; *IQR* interquartile range.

^a^Median (IQR) or Frequency (%).

^b^Wilcoxon rank sum test; Pearson’s Chi-squared test.

**Fig. 1:**
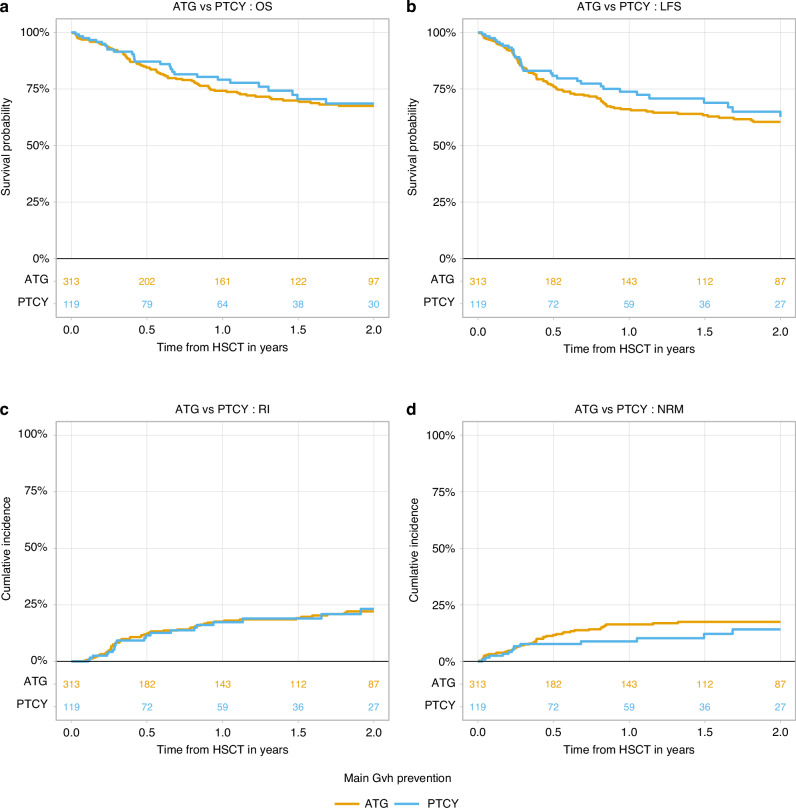
Treatment outcomes. **a** Overall survival (OS), **b** leukemia-free survival (LFS), **c** relapse incidence (RI) and **d** non-relapse mortality (NRM). HSCT hematopoietic stem cell transplantation, ATG anti-thymocyte globulin, PTCY post-transplant cyclophosphamide.

Median follow-up was 1.9 years (95% CI: 1.5–2.1) in the ATG group and 1.2 years (95% CI: 1.1–1.8) in the ptCy group. Two-year overall survival (OS) was similar: 67.5% (95% CI: 61.0–73.2) for ATG and 68.6% (95% CI: 56.7–77.8) for ptCy. Leukemia-free survival (LFS) at 2 years was 60.4% (95% CI: 53.8–66.4) for ATG and 62.6% (95% CI: 50.4–72.6) for ptCy. Relapse incidence (RI) at 2 years was 22.1% (95% CI: 17.0–27.7) for ATG and 23.2% (95% CI: 14.3–33.4) for ptCy. Non-relapse mortality (NRM) was 17.5% (95% CI: 13.1–22.4) and 14.1% (95% CI: 7.5–22.8) in ATG and ptCy groups, respectively. Transplant outcomes including hazard ratios are summarized in Table 2 and Fig. 1.

**Table 2:** Transplant outcomes—estimates and univariate Cox model results.

Outcome	All patients	ATG	ptCY	Univariate HR	P-value
OS (2 y) %	67.9% (62.4–72.9)	67.5% (61–73.2)	68.6% (56.7–77.8)	0.87 (0.59–1.3)	0.498
LFS (2 y)	61.2% (55.5–66.4)	60.4% (53.8–66.4)	62.6% (50.4–72.6)	0.86 (0.6–1.24)	0.424
RI (2 y)	22.4% (17.9–27.2)	22.1% (17–27.7)	23.2% (14.3–33.4)	1.01 (0.65–1.56)	0.976
NRM (2 y)	16.4% (12.7–20.6)	17.5% (13.1–22.4)	14.1% (7.5–22.8)	0.7 (0.38–1.29)	0.253
aGVHD ≥ II (180 d)	25.4% (21.3–29.8)	23.7% (19–28.8)	29.7% (21.7–38.1)	1.28 (0.88–1.85)	0.199
aGVHD ≥ III (180 d)	9.8% (7.1–12.9)	8.9% (6–12.6)	11.9% (6.8–18.5)	1.37 (0.73–2.58)	0.321
cGVHD (2 y)	29.8% (24.7–35.1)	30.4% (24.5–36.5)	28.3% (18.5–38.9)	0.75 (0.48–1.18)	0.212
extcGVHD (2 y)	12.4% (9–16.5)	12.1% (8.2–16.7)	13.6% (7–22.5)	1.06 (0.56–1.99)	0.859

Estimates and hazard ratios are shown with 95% confidence intervals.

*OS* overall survival, *LFS* leukemia-free survival, *RI* relapse incidence, *NRM* non-relapse mortality, *aGVHD* acute graft-versus-host disease, *cGVHD* chronic graft-versus-host disease, *extcGVHD* extensive chronic graft-versus-host disease, *d* days, *y* years.

## Discussion

Our findings indicate that long-term outcomes of allo-HCT in patients with comorbidities associated with cardiovascular risk are not inferior when ptCy is used instead of ATG as the backbone of GVHD prophylaxis. The main strengths of our study are the inclusion of a relatively large and homogeneous cohort of patients with AML in first complete remission, use of propensity score matching to balance groups for main risk factors known to influence transplant outcomes as well as exclusion of patients transplanted from haploidentical or 8/10 HLA mismatched unrelated donors, as results of ptCY and ATG prophylaxis are not equivalent in these patients. Primary limitations of this analysis include the absence of data on early cardiac events (ECE), as such information is not captured in the registry dataset, and the retrospective, non-randomized design of the study. Consequently, we cannot exclude the possibility that patients with a more significant cardiovascular history were selectively excluded from receiving ptCy. Furthermore, the data were based on pretransplant HCT-comorbidity index reports, which may not represent an adequate screening tool for cardiovascular risk in this context as known risk factors for cardiac events (e.g. dyslipidemia, smoking, hypertension) are not recorded. Data on exact schedule and dosing of ptCy were also not available.

Several single center and one multicentric retrospective study of cardiac events after ptCy have been published [4–6]. In these studies, ptCy was associated with an increased risk of early cardiac events, without worsening of long-term outcomes. Cardiac events were associated with increased long-term mortality. Our results are in agreement with these reports.

LeMaistre et al. [7] analyzed results of nonmyeloablative transplants with ptCy in patients with reduced systolic function. While the number of patients transplanted with systolic dysfunction in this analysis was small, overall results show feasibility of nonmyeloablative transplant with ptCy in this population.

Cyclophosphamide cardiotoxicity is caused by its metabolites, mainly acrolein, through oxidative stress, inflammation, and endothelial dysfunction. Cyclophosphamide metabolism and elimination is influenced by the dosing regimen, the enzyme CYP3A4 and aldehyde dehydrogenase activity and renal function, leading to large inter-individual variability in plasma levels of parent compound, levels of active metabolite (phosphoramide mustard) and acrolein, which is responsible for toxic effects [8, 9]. More data are needed on the interplay between preexisting comorbidities, conditioning regimen, organ function, cyclophosphamide pharmacokinetics and other determinants of long-term outcomes [10].

In conclusion, our study suggests that the use of ptCy for GVHD prophylaxis in patients with cardiovascular comorbidities yields long-term outcomes comparable to those observed with ATG-based approaches. While these findings support the feasibility of ptCy in this population, the absence of early cardiac event data and the retrospective nature of the analysis underscore the need for prospective studies. Future research should focus on better cardiovascular risk stratification, the development and validation of a screening tool specific for ptCy cardiotoxicity, and evaluation of cyclophosphamide pharmacokinetics to identify patients at increased risk of cardiotoxicity. Such efforts will be essential to optimize patient selection and improve the safety of transplantation strategies involving ptCy.

## Data Availability

The final analysis dataset will be available upon specific request to the Working Party Chair.
